# Effect of previous and current vaccination against influenza A(H1N1)pdm09, A(H3N2), and B during the post-pandemic period 2010-2016 in Spain

**DOI:** 10.1371/journal.pone.0179160

**Published:** 2017-06-14

**Authors:** Alin Gherasim, Iván Martínez-Baz, Jesús Castilla, Francisco Pozo, Amparo Larrauri, the cycEVA working group

**Affiliations:** 1 National Epidemiology Centre, Carlos III Health Institute, Madrid, Spain; 2 Instituto de Salud Pública y Laboral de Navarra, IdiSNA—Navarra Institute of Health Research, Pamplona, Spain; 3 CIBER de Epidemiología y Salud Pública (CIBERESP), Institute of Health Carlos III, Madrid, Spain; 4 National Centre of Microbiology, National Influenza Reference Laboratory, WHO-National Influenza Centre, Carlos III Health Institute, Madrid, Spain; University of Calgary, CANADA

## Abstract

**Background:**

Recent studies suggest that the protective effect of the current influenza vaccine could be influenced by vaccination in previous seasons. We estimated the combined effect of the previous and current influenza vaccines from the 2010–2011 season to the 2015–2016 season in Spain.

**Methods:**

We performed a test-negative case-control study in patients ≥9 years old. We estimated the influenza vaccine effectiveness (IVE) against influenza A(H1N1)pdm09, A(H3N2), and B virus.

**Results:**

We included 1206 influenza A(H1N1)pdm09 cases, 1358 A(H3N2) cases and 1079 B cases. IVE against A(H1N1)pdm09 virus in the pooled-season analysis was 53% (95% Confidence Interval (CI): 21% to 72%) for those vaccinated only in the current season and 50% (95%CI: 23% to 68%) for those vaccinated in the both current and previous seasons. Against the influenza A(H3N2) virus, IVE was 17% (95%CI: -43% to 52%) for those vaccinated only in the current season and 3% (95%CI: -33% to 28%) for those vaccinated in both seasons. Regarding influenza B, we obtained similar IVEs for those vaccinated only in the current and those vaccinated in both seasons: 57% (95%CI: 12% to 79%) and 56% (95%CI: 36% to 70%), respectively.

**Conclusion:**

Our results suggested no interference between the previous and current influenza vaccines against A(H1N1)pdm09 and B viruses, but a possible negative interference against A(H3N2) virus.

## Introduction

Influenza represents a public health problem, therefore it is generally recommended that population groups at risk of severe complications or death are vaccinated: the elderly (above 64 years old), those younger than 65 with chronic conditions, pregnant women or persons at risk due to their profession [1; 2].The influenza vaccine is the main preventive measure available, but as influenza viruses undergo frequent changes in their surface antigens, it needs re-formulation each year, with the aim of antigenically matching the circulating strains [3].

Since the 2008–2009 season, have estimated the influenza vaccine effectiveness (IVE) in Spain using the cycEVA study (case-control study for the influenza vaccine effectiveness in Spain), which is the Spanish component of the I-MOVE project for monitoring the IVE in Europe. Ever since, within the cycEVA study we have continuously employed a case-control test-negative design. This is a method that is used worldwide as one of the most appropriate to estimate IVE, because it minimizes the habitual biases of observational studies [4–6].

A series of factors influenced the yearly-observed IVE estimates. Apart from the antigenic match with the vaccine-circulating strain, recent studies have suggested that the protective effect of the current season´s influenza vaccinecould be also influenced by influenza vaccination in previous seasons (N.B. This article refers to “current” in the sense of the season under analysis rather than the present moment). While several papers did not detect any evidence for decreasing protection with repeated vaccination [7;8], other authors described an effect between the previous season´s vaccination and the current one. This was observed in a single season or across various seasons, with some studies suggesting a negative interference between the previous and the current season´s influenza vaccine [9–12].

We present the IVE estimates for the current season´s influenza vaccine, as well as the combined effect of the previous and current vaccine, as obtained during six post-pandemic seasons in Spain, 2010–2011 to 2015–2016, with the cycEVA study. We also interpret these results in the context of the similarities between the circulating strains and vaccine strains, both in current and previous season.

## Methods

### Study setting, design and data collection

We have used data obtained from the cycEVA study, from the 2010–2011 to 2015–2016 influenza seasons. The cycEVA study is an observational case-control study to monitor influenza vaccine effectiveness (IVE) in Spain, which is conducted within the framework of the Spanish Influenza Sentinel Surveillance System (SISSS)[13]. During the study period, between five and eight out of the 17 regional sentinel networks participated each season.

The methodology of the cycEVA study has been described previously in the literature [14;15]. Briefly, following a common European protocol [16], sentinel practitioners (SPs) reported cases of influenza-like illness (ILI) on a weekly basis according to a definition that is based on the European Commission (EU) ILI case definition [17]. They systematically swabbed patients below 65 years old (the first two patients with influenza-like illness (ILI) who had consulted a sentinel physician each week) and all patients above 64 years old. For each recruited patient, the SPs collected information on demographic data (age, sex, sentinel network), previous and current vaccination status (including date of vaccination) and presence of a chronic condition (i.e., chronic cardiovascular, pulmonary, hepatic or renal diseases, congenital or acquired immunodeficiency and diabetes mellitus), as well as pregnancy status, and obesity (defined as a body mass index BMI ≥40 kg/m^2^).

For all six seasons, we used a case-control test-negative design. We defined cases as ILI patients who had a reverse transcription polymerase chain-reaction (RT-PCR) and/or cell culture test, positive for influenza (A(H1N1)pdm09, A(H3N2) or B); whereas controls tested negative for all influenza viruses. In each season, the study started when a sporadic circulation of influenza viruses was detected in the participant surveillance networks. We considered a patient vaccinated if he/she had received the trivalent seasonal influenza vaccine at least 14 days before the onset of ILI symptoms; patients receiving the influenza vaccine less than 14 days before symptoms’ onset were considered unvaccinated, whereas those without a vaccination date were excluded from the analysis.

### Data analysis

Since the objectives of this work are different from those of previous cycEVA studies [18–21], we reanalyzed data within cycEVA using the same analysis approach across all six seasons studied. We have considered only patients of nine years and above, since below this age complete vaccination status might include vaccination in the previous season; we also restricted the analysis to the ILI patients swabbed less than eight days since the onset of symptoms. For each season, we estimated the IVE against the predominant influenza virus (≥60% of the total type/subtype of influenza virus circulation), or against viruses with a circulation of at least 25% of the total influenza viruses, during the period with continuous circulation of that specific type/subtype. We estimated the IVE for all those aged nine and above, the target groups for influenza vaccination; and, in a further sensitivity analysis, we restricted the analysis to those swabbed four days or less since onset of symptoms. We considered target groups as all those patients above 64 years old (60 in some sentinel networks), those with a chronic condition or other risk factor for influenza (obesity or pregnancy), or who met any other criteria to be vaccinated in Spain (such as healthcare workers or caregivers). We explored the distribution of the covariates between cases and controls using a χ^2^ test. We used logistic regression models to evaluate the odds ratios (OR) with 95% confidence intervals (95%CI), adjusting for age-groups (9–14, 15–44, 45–64, ≥65 years), sex, chronic condition, sentinel network and week of swabbing, as well as influenza season in the pooled analysis. We estimated the IVE with the formula (1-OR)x100 for vaccination.

In the main study analysis, we estimated the effect of previous and current seasons’ vaccination, against considered virus type/subtype, in pooled analysis and by each season for all study subjects. In a further refinement, we restricted the analysis to those belonging to the target groups. In the 2010–2011 influenza season, the pandemic vaccine was considered as a previous vaccination against A(H1N1)pdm09 for the purposes of the IVE. To determine current vaccination status we used the available vaccination date, while for patients vaccinated in the previous season we had only yes/no information. The effects of previous and current vaccinations were evaluated using four categories: vaccinated only in the previous season, vaccinated only in the current season, receiving both vaccines, and unvaccinated. We estimated the adjusted IVE with the 95%CI for each category, adjusting for the same variables as in the primary analysis and using the unvaccinated as a reference. Differences in the IVE between those vaccinated only in the “current” season and those receiving both vaccines were explored, using those vaccinated only in the current season as reference.

For each season we have provided the results on the genetic characterization of influenza strains, from cases notified at the national level in Spain. Strains were genetically characterized at the World Health Organization (WHO) National Influenza Centre in Madrid (Spain), by sequencing the HA1 fragment of the viral hemagglutinin gene.

## Results

### Influenza seasons and characteristics of cases and controls

**Fig 1:**
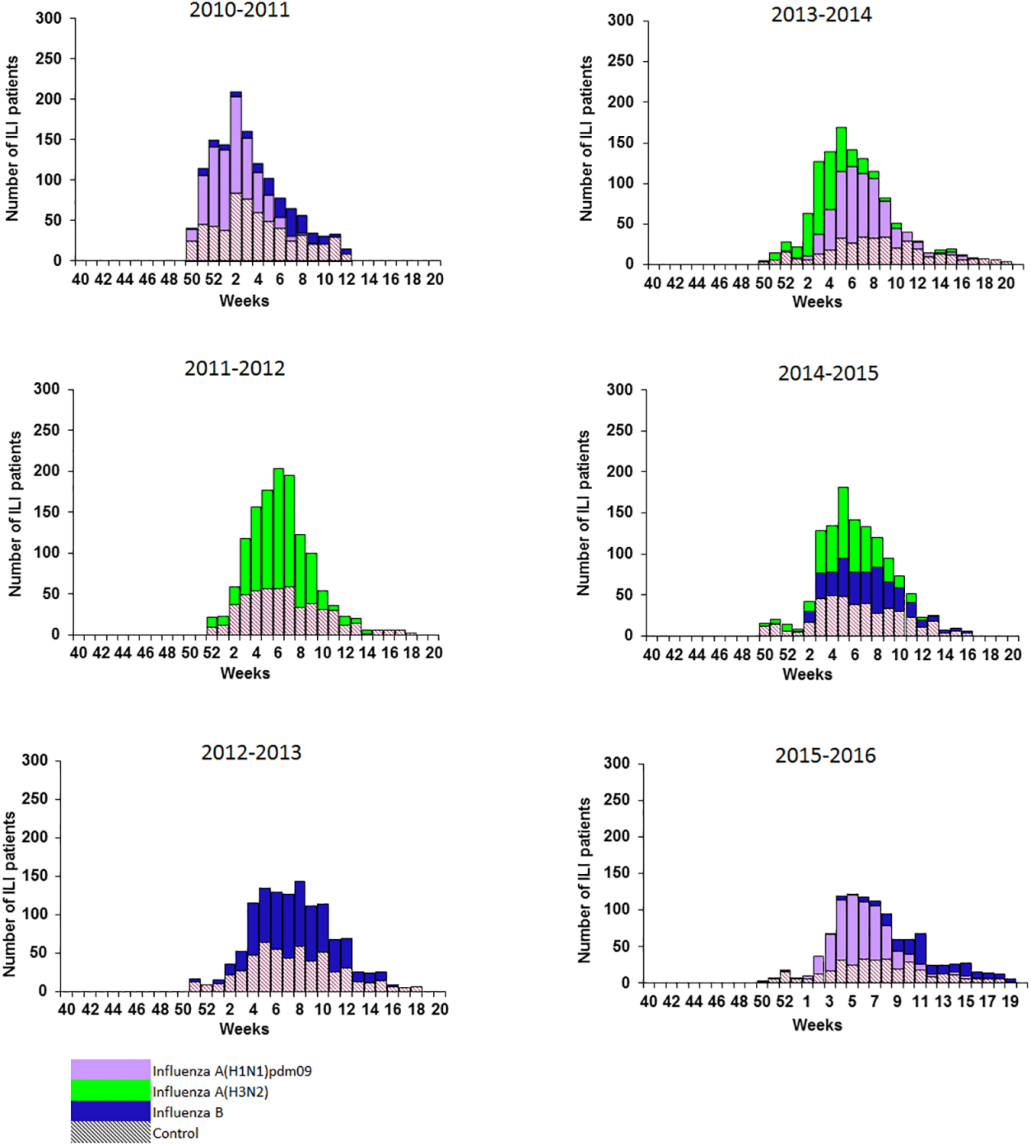
Cases and controls recruitment, cycEVA study, seasons 2010–2011 to 2015–2016, Spain.

According to the seasonal distribution of recruited influenza A(H1N1)pdm09, A(H3N2) and B cases, IVE was estimated against influenza A(H1N1)pdm09 in 2010–2011, 2013–2014 and 2015–2016 seasons; against influenza A(H3N2) in the 2011–2012, 2013–2014 and 2014–2015; and against influenza B in 2010–2011, 2012–2013, 2014–2015 and 2015–2016 seasons (Fig 1).

A total of 507, influenza A(H1N1)pdm09 cases were included for the 2010–2011 season, 303 for 2013–2014 and 396 for 2015–2016, respectively. There were less cases than controls belonging to target groups for vaccination in all A(H1N1)pdm09 influenza seasons. Compared to the controls, cases were less vaccinated in the current, as well as in the previous season (Table 1).

**Table 1:** Characteristics of influenza A(H1N1)pdm09 cases and test negative controls in Spain, seasons 2010–2011, 2013–2014 and 2015–2016.

Influenza season	2010–2011			2013–2014			2015–2016		
	Controls	Cases		Controls	Cases		Controls	Cases	
	N (%)	N (%)	p	N (%)	N (%)	p	N (%)	N (%)	p
**Total**	**443 (100)**	**507 (100)**		**444 (100)**	**303 (100)**		**265 (100)**	**396 (100)**	
**Age groups (years)**			< .001			.110			.006
9–14	51 (12)	50 (10)		50 (11)	33 (11)		36 (14)	32 (8)	
15–44	252 (57)	321 (63)		222 (50)	165 (54)		131 (49)	191 (48)	
45–64	96 (22)	119 (24)		121 (27)	86 (28)		69 (26)	144 (36)	
≥65	44 (10)	17 (3)		51 (11)	19 (7)		29 (11)	29 (7)	
**Sex**			.448			.332			.579
Male	218 (49)	237 (47)		221 (50)	162 (53)		139 (52)	199 (50)	
Female	225 (51)	270 (53)		223 (50)	141 (47)		126 (48)	197 (50)	
**Target groups**			.005			.033			.012
No	304 (69)	389 (77)		302 (68)	228 (75)		175 (66)	297 (75)	
Yes	139 (31)	118 (23)		142 (32)	75 (25)		90 (34)	99 (25)	
**Major chronic conditions**			.209			.052			.080
No	305 (83)	360 (86)		345 (78)	253 (84)		199 (75)	320 (81)	
Yes	63 (17)	58 (14)		99 (22)	50(16)		66 (25)	76 (19)	
**Previous influenza vaccine**^**a**^			< .001			.007			.055
No	390 (93)	492 (98)		384 (86)	281 (93)		229 (86)	360 (91)	
Yes	33 (7)	9 (2)		60 (14)	22 (7)		36 (14)	35 (9)	
**Current influenza vaccine**			< .001			.012			.001
No	388 (88)	485 (96)		386 (87)	281 (93)		223 (84)	366 (92)	
Yes	55 (12)	22 (4)		58 (13)	22 (7)		42 (16)	30 (8)	
**Onset-swabbing ≤ 4days**			.057			.541			.736
No	18 (4)	10 (2)		10 (2)	9 (3)		5 (2)	9 (2)	
Yes	425 (96)	497 (98)		434 (98)	294 (97)		260 (98)	387 (98)	

^a^For the influenza season 2010–2011, the pandemic vaccine was considered as a previous vaccine.

A total of 674 A(H3N2) cases were included for the 2011–2012 season, 322 for 2013–2014 and 362, for 2014–2015 influenza season. Cases were older than controls in all A(H3N2) seasons, and for the 2011–2012 season, cases included more patients within the target groups than negative controls (Table 2).

**Table 2:** Characteristics of influenza A(H3N2) cases and test negative controls in Spain, seasons 2011–2012, 2013–2014 and 2014–2015.

Influenza season	2011–2012			2013–2014			2014–2015		
	Controls	Cases		Controls	Cases		Controls	Cases	
	N (%)	N (%)	p	N (%)	N (%)	p	N (%)	N (%)	p
**Total**	**430 (100)**	**674 (100)**		**440 (100)**	**322 (100)**		**358 (100)**	**362 (100)**	
**Age groups (years)**			.016			.876			.003
9–14	53 (12)	83 (12)		50 (11)	32 (10)		56 (16)	84 (23)	
15–44	223 (52)	301 (45)		220 (50)	159 (49)		160 (45)	158 (44)	
45–64	116 (27)	191 (28)		120 (27)	90 (28)		110 (31)	76 (21)	
≥65	38 (9)	99 (15)		50 (11)	41 (13)		32 (9)	44 (12)	
**Sex**			.788			.542			.374
Male	209 (49)	322 (48)		217 (49)	166 (52)		178 (50)	168 (46)	
Female	221 (51)	352 (52)		223 (51)	156 (48)		180 (50)	194 (54)	
**Target groups**			.023			.817			.866
No	320 (75)	461 (68)		300 (68)	217 (67)		267 (75)	268 (74)	
Yes	108 (25)	213 (32)		140 (32)	105 (33)		91 (25)	94 (26)	
**Major chronic conditions**			.836			.421			.981
No	365 (85)	569 (84)		343 (78)	243 (75)		291 (81)	294 (81)	
Yes	65 (15)	105 (16)		97 (22)	79 (25)		67 (19)	68 (19)	
**Previous influenza vaccine**			.805			.706			.208
No	374 (87)	581 (86)		380 (86)	275 (85)		324 (91)	317 (88)	
Yes	56 (13)	91 (14)		60 (14)	47 (15)		34 (9)	45 (12)	
**Current influenza vaccine**			.517			.424			.531
No	371 (86)	572 (85)		382 (87)	273 (85)		317 (89)	315 (87)	
Yes	59 (14)	102 (15)		58 (13)	49 (159		41 (11)	47 (13)	
**Onset-swabbing ≤ 4days**			.062			.697			.197
No	18 (4)	15 (2)		10 (2)	6 (2)		7 (2)	3 (1)	
Yes	412 (96)	659 (98)		430 (98)	316 (98)		351 (98)	359 (99)	

Regarding influenza B, in the 2010–2011 a total of 127 cases were included in the analysis, 512 in 2012–2013, 301 in 2014–2015 and 139 in 2015–2016 seasons, , and, respectively. In the 9–14 years age-group there were more cases than controls in the 2010–2011, 2012–2013 and 2015–2016 seasons. Overall, in comparison with the controls, cases were less vaccinated in the seasons 2012–2013 and 2015–2016, whereas previous vaccination showed a statistically significant difference only for the 2012–2013 season (Table 3).

**Table 3:** Characteristics of influenza B cases and test negative controls in Spain, seasons 2010–2011 to 2015–2016.

Influenza season	2010–2011			2012–2013			2014–2015			2015–2016		
	Controls	Cases		Controls	Cases		Controls	Cases		Controls	Cases	
	N (%)	N (%)	p	N (%)	N (%)	p	N (%)	N (%)	p	N (%)	N (%)	p
**Total**	**489 (100)**	**127 (100)**		**435 (100)**	**512 (100)**		**345 (100)**	**301 (100)**		**283 (100)**	**139 (100)**	
**Age groups (years)**			< .001			.014			.806			< .001
9–14	57 (12)	43 (34)		64 (15)	104 (20)		54 (16)	44 (15)		40 (14)	45 (32)	
15–44	272 (56)	58 (46)		221 (51)	219 (43)		157 (46)	129 (43)		136 (48)	63 (45)	
45–64	113 (23)	14 (11)		110 (25)	153 (30)		106 (31)	103 (34)		75 (27)	21 (15)	
≥65	47 (10)	12 (9)		40 (9)	36 (7)		28 (8)	25 (8)		32 (11)	10 (7)	
**Sex**			.138			.512			.518			.430
Male	244 (50)	73 (58)		220 (51)	248 (48)		175 (51)	145 (48)		150 (53)	68 (49)	
Female	245 (50)	54 (42)		215 (49)	264 (52)		170 (49)	156 (52)		133 (47)	71 (51)	
**Target groups**			.577			.515			.582			.014
No	330 (67)	89 (70)		306 (70)	370 (72)		256 (74)	229 (76)		187 (66)	108 (78)	
Yes	159 (33)	38 (30)		129 (30)	142 (28)		89 (26)	72 (24)		96 (34)	31 (22)	
**Major chronic conditions**			.389			.203			.189			.067
No	338 (83)	84 (79)		338 (78)	415 (81)		280 (81)	256 (85)		214 (76)	116 (83)	
Yes	70 (17)	22 (21)		97 (22)	97 (19)		65 (19)	45 (15)		69 (24)	23 (17)	
**Previous season influenza vaccination**^**a**^			.806			.001			.386			.088
No	433 (93)	116 (94)		379 (87)	487 (94)		312 (90)	278 (92)		244 (86)	126 (92)	
Yes	33 (7)	8 (6)		56 (13)	33 (6)		33 (10)	23 (8)		39 (14)	11 (8)	
**Current season influenza vaccination**			.088			< .001			.051			.003
No	428 (88)	118 (93)		377 (87)	480 (94)		304 (88)	279 (93)		238 (84)	131 (94)	
Yes	61 (12)	9 (7)		58 (13)	32 (6)		41 (12)	22 (7)		45 (16)	8 (6)	
**Onset-swabbing ≤ 4days**			.129						.103			.980
No	22 (5)	2 (2)		19 (4)	22 (4)	.957	8 (2)	14 (5)		6 (2)	3 (2)	
Yes	467 (95)	125 (98)		416 (96)	490 (96)		337 (98)	287 (95)		277 (98)	136 (98)	

^a^For the influenza season 2010–2011, the previous vaccination was considered the administration of the pandemic vaccine

### Influenza vaccine effectiveness results

The adjusted IVE against A(H1N1)pdm09 varied between 39% (95%CI: -13% to 67%) in 2013–2014 and 52% (95%CI: 20% to 78%) in 2015–2016 for all population. The pooled analysis revealed an IVE against A(H1N1)pdm09 of 50% (95% CI: 29% to 65%) and 42% (95%CI: 14% to 61%) for all population and target groups, respectively (Table 4).

**Table 4:** Influenza vaccine effectiveness in preventing laboratory-confirmed influenza by virus type/subtype in Spain, seasons 2010–2011 to 2015–2016.

Type/Subtype	Season/Type of analysis	Cases vaccinated/Total(%)	Controls vaccinated/Total(%)	Crude VE % (95%CI)	Adjusted VE^a^ (95%CI)
**A(H1N1)pdm09**	**2010–2011**				
	All population	22/507 (4)	55/443 (12)	68 (47; 81)	49 (1; 73)
	Target groups	19/118 (16)	49/139 (35)	65 (36; 81)	36 (-32; 69)
	Onset-swabbing ≤ 4 days	21/497 (4)	49/425 (12)	66 (43; 80)	50 (1; 74)
	**2013–2014**				
	All population	22/303 (7)	58/444 (13)	48 (13; 69)	39 (-13; 67)
	Target groups	19/75 (25)	54/142 (36)	45 (-3; 70)	40 (-21; 70)
	Onset-swabbing ≤ 4 days	20/294 (7)	58/434 (13)	53 (19; 72)	44 (-6; 70)
	**2015–2016**				
	All population	30/396 (8)	42/265 (16)	56 (28; 73)	52 (20; 78)
	Target groups	27/99 (27)	36/90 (40)	44 (-4; 69)	48 (-9; 75)
	Onset-swabbing ≤ 4 days	29/387 (8)	41/260 (16)	57 (28; 74)	59 (20; 79)
	**Pooled analysis**				
	All population	74/1206 (6)	155/1152 (14)	58 (44; 68)	50 (29; 65)
	Target groups	65/292 (22)	139/371 (38)	52 (32; 66)	42 (14; 61)
	Onset-swabbing ≤ 4 days	70/1178 (6)	148/1119 (13)	59 (44; 69)	51 (29; 66)
**A(H3N2)**	**2011–2012**				
	All population	102/674 (15)	59/430 (14)	-12 (-59; 21)	29 (-11; 55)
	Target groups	83/213 (39)	44/108 (41)	7 (-49; 42)	39 (-14; 67)
	Onset-swabbing ≤ 4 days	99/659 (15)	54/412 (13)	-17 (-68; 18)	28 (-14; 55)
	**2013–2014**				
	All population	49/322 (15)	58/440 (13)	-18 (-78; 22)	-18 (-104; 31)
	Target groups	40/105 (38)	54/140 (39)	2 (-65; 42)	-1 (-93; 47)
	Onset-swabbing ≤ 4 days	45/316 (14)	58/430 (14)	-7 (-62; 30)	-7 (-87; 39)
	**2014–2015**				
	All population	47/362 (13)	41/358 (12)	-15 (-80; 26)	-15 (-101; 34)
	Target groups	39/94 (42)	35/91 (39)	-14 (-104; 37)	-5 (-106; 47)
	Onset-swabbing ≤ 4 days	47/359 (13)	39/351 (11)	-21 (-90; 23)	-21 (-111; 31)
	**Pooled analysis**				
	All population	198/1358 (15)	158/1228 (13)	-15 (-45; 8)	4 (-28; 28)
	Target groups	162/412 (39)	133/339 (39)	0 (-35; 25)	10 (-27; 37)
	Onset-swabbing ≤ 4 days	191/1334 (14)	151/1193 (13)	-15 (-45; 8)	6 (-26; 30)
**B**	**2010–2011**				
	All population	9/127 (7)	61/489 (13)	46 (-11; 74)	63 (1; 86)
	Target groups	9/38 (24)	55/159 (35)	41 (-33; 74)	55 (-36; 85)
	Onset-swabbing ≤ 4 days	8/125 (6)	54/467 (12)	48 (-13; 76)	66 (4; 88)
	**2012–2013**				
	All population	32/512 (6)	58/435 (13)	57 (32; 72)	64 (37; 80)
	Target groups	31/142 (22)	47/129 (36)	51 (17; 71)	59 (22; 79)
	Onset-swabbing ≤ 4 days	26/490 (5)	54/416 (13)	62 (39; 77)	69 (44; 83)
	**2014–2015**				
	All population	22/301 (7)	41/345 (12)	41 (-1; 66)	43 (-6; 69)
	Target groups	17/72 (24)	34/89 (38)	50 (0; 75)	47 (-13; 75)
	Onset-swabbing ≤ 4 days	20/287 (7)	39/337 (12)	43 (-1; 67)	45 (-5; 71)
	**2015–2016**				
	All population	8/139 (6)	45/283 (16)	68 (29; 85)	55 (-17; 82)
	Target groups	7/31 (23)	38/96 (40)	55 (-14; 82)	29 (-132; 78)
	Onset-swabbing ≤ 4 days	7/136 (5)	44/277 (16)	71 (34; 87)	59 (-9; 85)
	**Pooled analysis**				
	All population	71/1079 (7)	205/1552 (13)	54 (39; 65)	57 (40; 70)
	Target groups	64/283 (23)	174/473 (37)	50 (30; 64)	66 (34; 70)
	Onset-swabbing ≤ 4 days	61/1038 (6)	191/1497 (13)	57 (42; 68)	62 (45; 73)

VE: Vaccine Effectiveness; CI: Confidence Intervals

^a^Adjusted VE by: age-groups (9–14; 15–44; 45–64; >64 years), sex, chronic conditions, sentinel network, week of swabbing and season (for the pooled analysis).

Against influenza A(H3N2), the adjusted IVE for all population was 29% (95%CI: -11% to 55%) in the 2011–2012 season; negative IVE point estimates were obtained in the 2013–2014 and 2014–2015. The pooled IVE point estimate against A(H3N2) was of 4% (95%CI: -28% to 28%) and 10% (95%CI: -27% to 37%) for the all population and target groups respectively (Table 4).

The adjusted IVE against influenza B, for all population, varied between 43% (95%CI: -6% to 69%) and 64% (95%CI: 37% to 80%) in 2014–2015 and 2012–2013 seasons, respectively. The pooled IVE against influenza B was 66% (95%CI: 34% to 70%) and 57% (95%CI: 40% to 70%), for the target groups and all population, respectively (Table 4).

When restricting the analysis to the patients swabbed 4 days or less after the onset of symptoms, we obtained similar results compared to all population analysis (Table 4).

### Previous and current vaccination analysis

IVE against A(H1N1)pdm09 showed a null or low adjusted estimate for patients receiving only the previous vaccination, with the exception of the 2010–2011 season where patients had received the previous monovalent pandemic vaccine: 65% (95%CI: -13% to 89%) (Table 5).

**Table 5:** Effect of current and previous influenza vaccination in patients ≥9 years by virus type/subtype in Spain, seasons 2010–2011 to 2015–2016.

Type/ subtype	Season	Vaccine and circulating strains			Unvaccinated	Vaccinated previous season only		Vaccinated current season only		Vaccinated both seasons	
		Previous season vaccine strain	Current season vaccine strain	Main Circulating strain	Cases/ Controls	Cases/ Controls	VE^a^ (95%CI)	Cases/ Controls	VE^a^ (95%CI)	Cases/ Controls	VE^a^ (95%CI)
**A(H1N1)pdm09**	2010–2011	A/California/7/2009	A/California/7/2009	A/California/7/2009^b^	474/355	5/11	65 (-13; 89)	18/35	45 (-11; 73)	4/19	73 (1; 93)
	2013–2014	A/California/7/2009	A/California/7/2009	A/StPetersburg/27/2011^b^	276/376	5/10	20 (-147; 74)	5/8	0 (-234; 70)	17/50	46 (-5; 73)
	2015–2016	A/California/7/2009	A/California/7/2009	A/SouthAfrica/3626/2013^b^	356/219	9/4	-55 (-446; 66)	4/10	74 (11; 92)	26/32	49 (-5; 75)
	Pooled analysis				1106/950	19/25	26 (-39; 61)	27/53	53 (21; 72)	47/101	50 (23; 68)
**A(H3N2)**	2011–2012	A/Perth/16/2009	A/Perth/16/2009	40%:A/England/259/2011^c^ 36%:A/Victoria/361/2011^c^ 22%: A/Iowa/19/2010^c^	564/364	8/7	45 (-55; 89)	17/10	14 (-107;65)	83/49	34 (-6; 59)
	2013–2014	A/Texas/50/2012	A/Texas/50/2012^d^	A/Texas/50/2012^b^	268/372	5/10	26 (-132; 77)	7/8	-1 (203;66)	42/50	-20 (-115;34)
	2014–2015	A/Texas/50/2012	A/Texas/50/2012	35% A/Samara/73/2013^b^ 50%A/HongKong/5738/2014^c^ 15%A/Switzerland/9715293/2013^c^	311/313	4/4	-29 (-507; 72)	6/11	45 (-59;80)	41/30	-45 (-171;21)
	Pooled analysis				1143/1049	17/21	23 (-51; 61)	30/29	17 (-43;52)	166/129	3 (-33; 28)
**B**	2010–2011	B/Brisbane/60/2008 (lin. Victoria)	B/Brisbane/60/2008 (lin. Victoria)	B/Brisbane/60/2008^b^ (lin. Victoria)	111/393	4/13	-62 (-544; 69)	2/14	51 (-182;91)	7/46	62 (-13; 87)
	2012–2013	B/Brisbane/60/2008 (lin. Victoria)	B/Wisconsin/1/2010 (lin. Yamagata)	B/Estonia/55669/2012^b^ B/Wisconsin/1/2010^b^ (lin. Yamagata)	472/372	8/5	-53 (-396; 53)	6/7	44 (-84; 83)	25/51	67 (40; 82)
	2014–2015	B/Massachusetts/02/2012 (lin. Yamagata)	B/Massachusetts/02/2012 (lin. Yamagata)	B/Phuket/3073/2013^b^ (lin. Yamagata)	275/314	4/4	-22 (-416; 71)	3/11	66 (-28; 91)	19/31	30 (-40; 64)
	2015–2016	B/Massachusetts/02/2012 (lin. Yamagata)	B/Phuket/3073/2013 (lin. Yamagata)	B/Brisbane/60/2008^c^ (lin. Victoria)	125/236	4/4	-151 (-1273; 54)	1/10	69 (-160; 96)	7/35	41 (-71; 79)
	Pooled analysis				983/1300	20/25	-54 (-200; 21)	12/42	57 (12; 79)	58/162	56 (36; 70)

VE: Vaccine Effectiveness; CI: Confidence Intervals; lin.: Lineage

^a^ VE: adjusted VE by: age-groups (9–14; 15–44; 45–64; >64), sex, sentinel network and week of swabbing and season (pooled analysis).

^b^ circulating strains antigenically matching the strain included in the current season vaccine

^c^ circulating strains antigenically miss-matched with the strain included in the current season vaccine

^d^ Similar to A/Victoria/361/2011

The IVE point estimates for patients receiving both current and previous vaccine were higher compared to those vaccinated only in the current season in the 2010–2011 and 2013–2014 seasons (p_comparison_ = .308 and .352, respectively), but lower in the 2015–2016 season (p_comparison_ = .321), although the differences were not significant. The pooled analysis revealed an IVE of 26% (95%CI: -39% to 61%) for those having received only the previous vaccination, and similar moderate values around 50% for those receiving either vaccines or only the current one (Table 5).

Against influenza A(H3N2), having received the previous season’s vaccine resulted in a moderate IVE of 45% (95%CI: -55% to 89%) in 2011–2012 season, and a lower or null value for the 2013–2014 and 2014–2015 seasons, respectively. The adjusted IVEs were 34% (95%CI: -6% to 59%) and 14% (95%CI: -107% to 65%) (p_comparison_ = .577) for those receiving both vaccines or only the current one, respectively, in the 2011–2012 season. However we found negative values for the season 2013–2014. For the 2014–2015 season, the IVE point estimate for both vaccines compared with only the current was lower: -45% (95%CI: -171% to 21%) vs 45% (95%CI: -59% to 80%) (p_comparison_ = .095). The pooled analysis revealed a low IVE of 3% (95%CI: -33% to 28%), for those receiving vaccinations in both seasons, and of 17% (95%CI: -43% to 52%) (p_comparison_ = .586) for those who received only the current vaccine (*Table 5*).

Regarding influenza B, we found negative IVE point estimates for patients receiving only the previous season’s vaccine. Having received both the previous and current season’s vaccines resulted in an IVE similar to those receiving only the current one, with point estimates slightly higher in two seasons, 2010–2011 and 2012–2013, and slightly lower in the other two seasons studied, 2014–2015 and 2015–2016, with no significant differences. The pooled analysis revealed negative IVE point estimates for those receiving only the previous season vaccine and similar, moderate IVE of 56% and 57%, for those receiving both vaccines and the current season vaccine only (*Table 5*).

When estimating the IVE against influenza A(H3N2), A(H1N1)pdm09 and B and restricting the analysis to the population group targeted for vaccination, we obtained similar results (“S1 Table”).

### Vaccine and circulating strains

The A(H1N1)pdm09 circulating strains for the three seasons included in the analysis matched the vaccine strain (Table 5).

In the three seasons with dominant or significant A(H3N2) circulation the previous season’s strain was similar to the current vaccine. The circulating strain matched the current season vaccine in 2013–2014 but only partially in 2014–2015, when 35% of the characterized strains belonged to A/Samara/73/2013 group, antigenically matching the A/Texas/50/2012 vaccine strain. 65% however, fell into two groups that had drifted from the A(H3N2) vaccine strain: A/HongKong/5738/2014 and A/Switzerland/9715293/2013. For the 2011–2012 season all circulating A(H3N2) strains were characterized as mismatched to the vaccine strain.

In all four seasons considered for IVE against influenza B, the previous and the current vaccine lineages were the same. Moreover they matched the current season’s circulating lineage. The only exception was the 2015–2016 season, when there was a mismatch between the current vaccine (Yamagata lineage) and the circulating strains (Victoria lineage) (Table 5).

## Discussion

Clarifying the combined effect of previous and current influenza vaccination remains a complex challenge; we have tried to disentangle this effect by using data across six influenza seasons in Spain for the firs time. To better understand the role of the previous year’s vaccine, we discuss our results taking also into account the match between the circulating and vaccine influenza strains.

Overall, we have found better vaccine effectiveness against confirmed A(H1N1)pdm09 and B influenza than against A(H3N2), similar results were obtained both for all patients included in our study as well as for the target groups for vaccination (Table 1).This was suggested both by the pooled analysis and for each influenza season under consideration. These results appear to be in line with previous studies performed in Spain and elsewhere, and confirm the usual poor performance of influenza vaccines against A(H3N2) [22–25].

When taking into account the previous vaccination, we have found some residual protective effect of the pandemic vaccine against 2010–2011 A(H1N1)pdm09 infection, that could be related with an adjuvant vaccine administered universally in Spain during the pandemic. Also we have seen that having received both the pandemic and the current vaccine, protected against A(H1N1)pdm09 infection in 2010–2011, an effect also described in previous studies [14;26;27]. Within each studied season, we did not find any interference between the previous season’s vaccine and the current vaccine’s performance, findings also confirmed by the pooled analysis. Along the same lines, a recently published study suggested an optimal vaccine protection against A(H1N1)pdm09 as individuals having received the current season’s vaccine and 1–2 prior doses [28]. These results could be explained by the relative genetic stability of the A(H1N1)pdm09 influenza virus since the pandemic, leading to the inclusion of the same vaccine strain A/California/7/2009 and a continued good match with strains circulating within in each of the three studied seasons [14;18;29].

The low or null IVE estimates obtained against A(H3N2) do not always appear to be in line with the degree of matching between the vaccine and circulating strains. In 2011–2012 and 2014–2015 seasons, a mismatch was described, whereas in 2013–2014 the vaccine did match the circulating strain; however the overall IVE remained suboptimal in all three seasons (Table 5). We registered the highest protective effect against A(H3N2) (34%) in the 2011–2012 season, the first post-pandemic influenza season dominated by A(H3N2) which had circulating strains discordant with the vaccine strain. We also found some residual protective effect of the previous season’s vaccine against A(H3N2) infection in the 2011–2012 and 2013–2014 seasons, but not in the 2014–2015 season. The current season’s vaccines showed suboptimal but slightly better protection against A(H3N2) for the vaccination-targeted population than in the general population (S1 Table), findings which are in line with a previous study [30]. While previous research described a poor correlation between the IVE and the circulating-vaccine A(H3N2) strain match [31], others studies have shown a correlation of the IVE with the match between circulating and vaccine strains [32–34]. When taking into account the previous vaccination, our results suggest a negative interference between the previous season’s vaccine and the current one for the 2014–2015 season. This effect was observed and described in the same season in Canada [35;36] and is consistent with the antigenic distance theory proposed by Smith & al. [37]. Indeed in Spain in the 2014–2015 season the strains predominantly circulating were A/HongKong/5738/2014 and A/Switzerland/9715293/2013 strains, which had antigenically drifted from the A/Texas/50/2012 vaccine strain, the same vaccine strain being used in the 2013–2014 season. The negative influence of the previous season’s vaccine in 2014–2015 could be explained by the small antigenic distance between the current and the previous vaccines and high antigenic distance between the 2014–2015 vaccine and the circulating strains. However, similar conditions were present for the 2011–2012 season in Spain, with the circulating A(H3N2) strains mismatched with the vaccine[38] and unchanged A(H3N2) vaccine strains in the 2011–2012 and 2010–2011 seasons, but without a clear suggestion of a negative effect from the previous vaccination. A question remains as to whether the antigenic distances between the respective circulating strains and the current vaccine strains were comparable in the two situations, or if other factors are behind the final IVE estimates against AH3N2 in the 2011–2012 season. Finally, the pooled analysis of the vaccine protection against A(H3N2) suggests some kind of interference between the previous season’s vaccine and current vaccine performance.

Regarding the protection against influenza B, the pooled analysis suggested no protective effect from the previous season’s vaccine, consistent with another similar study [39], and no negative interference between the previous season’s vaccine and current vaccine protection for all four studied seasons. By contrast, another study taking into account repeated vaccination over eight seasons in the United States found evidence of residual protection against influenza B [12]. For the 2010–2011 and 2012–2013 seasons, we have found no suggestion of negative interference between the previous and the current season’s vaccination; having received both vaccines resulted in moderate protective effect (around 65%) against confirmed Influenza B, significant for the 2012–2013 season and comparable to results described elsewhere [40]. For 2014–2015 and 2015–2016 seasons, a slightly decrease in IVE point estimates was registered for those receiving both vaccines compared to those having only the current, which was more pronounced for the 2014–2015 season. The small sample size and overlapping 95%CI however, do not allow strong conclusions to be drawn with respect to negative interference from the previous vaccination. In all but one influenza season, the circulating Influenza B strain matched the vaccine strain, partially explaining the moderate effectiveness of the vaccine. The 2015–2016 season was characterized by the discordance of circulating and vaccine lineages (Victoria and Yamagata, respectively), but the IVE was still moderate, suggesting possible cross-lineage protection, previously described in other settings [10;40].

In target groups for vaccination, the higher vaccine protection against A(H1N1)pdm09 and B was provided by vaccination in both previous and current seasons, whereas a possible interference between the previous and current vaccine was also observed against A(H3N2) (Table 5).

Our study has several limitations. The most important of which are the low vaccine coverage and small sample size, especially for the categories used when evaluating the previous vaccination effect, rendering imprecise estimations in some analysis, and reflected by wide confidence intervals. Moreover, for this analysis we could take into account the vaccination in the previous season, but we could not evaluate the repeated vaccination effect, since this information was not available. We discussed our results taking into account the match between circulating and vaccine strains, which was available for genetic characterization at national level but not in the cycEVA sentinel regions. Although differences in the distribution of circulating strains might exist, we consider these differences too small to significantly influence our results. We agree however with other researchers, however, that studies estimating the IVE against specific clade, with a randomized, and therefore unbiased selection of strains to be genetically characterized, would be of added value towards minimizing this limitation. Additionally, our study did not consider the effect that the use of several vaccine types might have on our results since it has varied by age group and Spanish sentinel network; however adjusting by these two variables might overcome this limitation.

In conclusion, our study results suggest an moderate protective effect against overall influenza A(H1N1)pdm09 and B, and a low vaccine effectiveness against A(H3N2). The pooled results of the studied seasons against predominant influenza type/subtype revealed no interference between the previous and current vaccine against A(H1N1)pdm09 and B and a possible negative interference against A(H3N2). Vaccine protection achieved against A(H1N1)pdm09 and B with the current vaccine or with both, the previous and current vaccine, is always superior to not being vaccinated. We have tried to explain these results by considering the previous vaccination effect and taking into account the match between circulating and vaccine strain. We have concluded that only the match between circulating and vaccine strains alone, cannot explain the obtained IVEs, and we underline the need of combined immunological and epidemiological studies, to better understand how these two elements are correlated.

## Supporting information

S1 Table Effect of current and previous influenza vaccination in patients ≥9 years belonging to target groups by virus type/subtype in Spain, seasons 2010–2011 to 2015–2016. VE: Vaccine effectiveness; CI: Confidence Intervals; lin: lineage.
a VE: adjusted VE by: age-groups (9–14; 15–44; 45–64; >64), sex, sentinel network and week of swabbing and season (pooled analysis).
b circulating strains antigenically matching the strain included in the current season vaccine.
c circulating strains antigenically miss-matched with the strain included in the current season vaccine. d Similar to A/Victoria/361/2011.
(DOCX)
