# Supplementary material for: Effect of previous and current vaccination against influenza A(H1N1)pdm09, A(H3N2), and B during the post-pandemic period 2010-2016 in Spain
Source: PLoS One. 2017 Jun 14;12(6):e0179160. doi: 10.1371/journal.pone.0179160 (PMC5470701; doi:10.1371/journal.pone.0179160)
Supplement: S1 Table — VE: Vaccine effectiveness; CI: Confidence Intervals; lin: lineage. a VE: adjusted VE by: age-groups (9–14; 15–44; 45–64; >64), sex, sentinel network and week of swabbing and season (pooled analysis). b circulating strains antigenically matching the strain included in the current season vaccine. c circulating strains antigenically miss-matched with the strain included in the current season vaccine. d Similar to A/Victoria/361/2011. (DOCX) [file pone.0179160.s001.docx]

**S1 Table. Effect of current and previous influenza vaccination in patients ≥9 years belonging to target groups by virus type/subtype in Spain, seasons 2010-2011 to 2015-2016**

| **Type/ subtype** | **Season** | **Vaccine and circulating strains** | | | **Unvaccinated** | **Vaccinated previous season only** | | **Vaccinated current season only** | | **Vaccinated both seasons** | |
| --- | --- | --- | --- | --- | --- | --- | --- | --- | --- | --- | --- |
|  |  | **Previous season vaccine strain** | **Current season vaccine strain** | **Main Circulating strain** | **Cases/**  **Controls** | **Cases/**  **Controls** | **VE^a^ (95%CI)** | **Cases/**  **Controls** | **VE^a^ (95%CI)** | **Cases/**  **Controls** | **VE^a^ (95%CI)** |
|  |  |  |  |  |  |  |  |  |  |  |  |
| **A(H1N1)pdm09** | 2010-2011 | A/California/7/2009 | A/California/7/2009 | A/California/7/2009^b^ | 96/80 | 3/9 | 82 (16; 96) | 16/31 | 28 (-59; 68) | 3/17 | 79 (8; 95) |
|  | 2013-2014 | A/California/7/2009 | A/California/7/2009 | A/StPetersburg/27/2011^b^ | 52/83 | 4/5 | -55 (-580; 64) | 4/8 | 13 (-231; 77) | 15/46 | 43 (-22; 74) |
|  | 2015-2016 | A/California/7/2009 | A/California/7/2009 | A/SouthAfrica/3626/2013^b^ | 67/50 | 4/4 | 31 (-240; 86) | 3/6 | 67 (-50; 93) | 24/30 | 47 (-19; 76) |
|  | Pooled analysis |  |  |  | 215/213 | 11/18 | 33 (-51; 70) | 23/45 | 37 (-14; 65) | 42/93 | 48 (15; 68) |
|  |  |  |  |  |  |  |  |  |  |  |  |
| **A(H3N2)** | 2011-2012 | A/Perth/16/2009 | A/Perth/16/2009 | 40%: /England/259/2011^c^ | 126/62 | 4/2 | 45 (-230; 91) | 14/8 | 31 (-97; 76) | 67/36 | 44 (-9; 71) |
|  |  |  |  | 36%: /Victoria/361/2011^c^ |  |  |  |  |  |  |  |
|  |  |  |  | 22%: A/Iowa/19/2010^c^ |  |  |  |  |  |  |  |
|  | 2013-2014 | A/Texas/50/2012 | A/Texas/50/2012 | A/Texas/50/2012^b^ | 63/81 | 2/5 | 41 (-266; 90) | 5/8 | 36 (-129; 82) | 35/46 | -7 (-117; 47) |
|  | 2014-2015 | A/Texas/50/2012 | A/Texas/50/2012 | 35% A/Samara/73/2013^b^ | 52/53 | 3/3 | 1 (-575; 86) | 4/9 | 65 (-40; 91) | 35/26 | -32 (-177; 37) |
|  |  |  |  | 50%A/HongKong/5738/2014^c^  15%/Switzerland/9715293/2013^c^ |  |  |  |  |  |  |  |
|  | Pooled analysis |  |  |  | 241/196 | 9/10 | 10 (-138; 66) | 23/25 | 28 (-35; 62) | 137/108 | 7 (-36; 36) |
|  |  |  |  |  |  |  |  |  |  |  |  |
| **B** | 2010-2011 | B/Brisbane/60/2008  (lin. Victoria) | B/Brisbane/60/2008  (lin. Victoria) | B/Brisbane/60/2008^b^  (lin. Victoria) | 28/92 | 1/11 | 59 (-311; 96) | 2/11 | 51 (-232; 93) | 7/43 | 61 (-31; 88) |
|  | 2012-2013 | B/Brisbane/60/2008  (lin. Victoria) | B/Wisconsin/1/2010  (lin. Yamagata) | B/Estonia/55669/2011^b^ B/Wisconsin/1/2010^b^  (lin. Yamagata) | 106/80 | 5/2 | -100 (-1184; 69) | 6/3 | -7 (-406; 77) | 24/44 | 63 (28; 81) |
|  | 2014-2015 | B/Massachusetts/02/2012 (lin. Yamagata) | B/Massachusetts/02/2012 (lin. Yamagata) | B/Phuket/3073/2013^b^  (lin. Yamagata) | 52/53 | 3/2 | -91 (-1166; 71) | 2/9 | 82 (4; 97) | 15/25 | 26 (-71; 68) |
|  | 2015-2016 | B/Massachusetts/02/2012 (lin. Yamagata) | B/Phuket/3073/2013  (lin. Yamagata) | B/Brisbane/60/2008^c^  (lin. Victoria) | 21/54 | 3/4 | -242 (-2405; 53) | 1/6 | 36 (-683; 95) | 6/32 | 9 (-239; 76) |
|  | Pooled analysis |  |  |  | 207/279 | 12/19 | -27 (-195; 45) | 11/29 | 50 (-13; 78) | 52/144 | 57 (33; 72) |
|  |  |  |  |  |  |  |  |  |  |  |  |

VE: Vaccine effectiveness; CI: Confidence Intervals; lin: lineage

^a^ VE: adjusted VE by: age-groups (9-14; 15-44; 45-64; >64), sex, sentinel network and week of swabbing and season (pooled analysis).

^b^ circulating strains antigenically matching the strain included in the current season vaccine

^c^ circulating strains antigenically miss-matched with the strain included in the current season vaccine

^d^ Similar to A/Victoria/361/2011
